# Complete Genome Sequence of Feline Calicivirus Strain GX01-2013 Isolated from Household Cats in Guangxi, Southern China

**DOI:** 10.1128/genomeA.00993-16

**Published:** 2016-09-22

**Authors:** Ping He, Huabo Zhou, Weifeng Zhao, Jianqiao He, Ruikai Li, Yangbao Ding, Quanjiang Chen, Lin Liu, Zuzhang Wei, Weijian Huang, Ying Chen

**Affiliations:** a College of Animal Science and Technology, Guangxi University, Nanning, China; b Huabo Pet Hospital, Nanning, China

## Abstract

Here, we report the complete genome of a feline calicivirus (FCV) originating from household cats in Guangxi, southern China, in September 2013. To understand its genetic characteristics, we isolated FCV strain GX01-2013 from MDCK cells and determined its complete genome sequence.

## GENOME ANNOUNCEMENT

Feline calicivirus (FCV), a member of the genus *Vesivirus* of the family *Caliciviridae*, is a highly infectious pathogen of cats which can cause upper respiratory tract disease (1). The FCV has a small, single-stranded positive-sense RNA genome that encodes three open reading frames (ORFs). ORF1 encodes nonstructural proteins, and ORF2 and ORF3 are located in the 3′-end of the genome and encode the capsid protein (VP1) and structural protein (VP2), respectively (2). Immune system pressure has been regarded as the most important reason for the appearance of newly emerging FCV strains, which has contributed to virulence difference and vaccine failures (3). Here, we describe the full-length genome sequence of FCV strain GX01-2013, which could replicate in MDCK cells effectively.

In this study, FCV strain GX01-2013 was isolated from household cats with severe respiratory symptoms, including conjunctivitis, nasal discharges, lethargy, and fever (39.3 to 41.5°C). The positive samples were treated by 200 U/ml penicillin, 200 mg/ml streptomycin, and 100 µg/ml gentamicin and centrifuged at 2,000 rpm for 5 min and run through a filter with 0.45-µm pores (Millipore, Bedford, MA, USA) to isolate the virus in MDCK cells. The virus was harvested at 48 h postinoculation for RNA extraction. cDNA was synthesized using an oligo(dT) primer. The 2.4-kb, 2.9-kb, and 2.3-kb overlapping cDNA fragments covering the complete genome were amplified with LA *Taq* DNA polymerase (TaKaRa) and cloned into the pMD18-T vector. The positive clone was sequenced by vector-universal primers and an FCV-specific primer by JET LI Biology (Shanghai, China).

The genome of FCV strain GX01-2013 consists of 7,704 nucleotides (nt), encoding three ORFs: ORF1 (nt 20 to 5311), ORF2 (nt 5314 to 7320), and ORF3 (nt 7317 to 7637). The 5′ and 3′untranslated regions were 19 nt and 67 nt long, respectively. The highest nucleotide sequence identity (82.1%) was to the genome of strain HRB-SS (accession no. KM016908), but the lowest identities were only 75.2% and 76%, respectively, with strains GD (accession no. GU214989) and FB-NJ-13 (accession no. KM111557) isolated from China. Phylogenic analysis showed that FCV strain GX01-2013 was grouped with the same cluster of HRB-SS, UTCVM-NH2 (accession no. AY560114), and FCV/DD/2006/GE (accession no. DQ424892). FCV strain GX01-2013 had only 78.5%, 78.7%, and 79.6% nucleotide homology with vaccine strains F9 (accession no. NC001481), FCV 2024 (accession no. AF479590), and FCV F4 (GenBank accession no. D31836), respectively. Interestingly, there was a complete conserved sequence of 15 nt (5′-TGCGCCTAACCCCAG-3′) in the 3′-end of FCV strains HRB-SS, 12Q087-1 (accession no. KJ572400), 12Q087-5 (accession no. KJ572401), and FCV CFI/68 (GenBank accession no. U13992). However, 57 nt were inserted into the 3′-end of the FCV strain GX01-2013 genome (from nt 7631 to nt 7687), differentiating it from these four FCV strains. Whether the inserted pattern in the 3′-end will affect the strain’s replication in MDCK cells or its virulence will require further study.

### Accession number(s).

The genome sequence of FCV strain GX01-2013 has been deposited in GenBank under the accession number KT970059.
